# Hidden surface microstructures on Carboniferous insect *Brodioptera sinensis* (Megasecoptera) enlighten functional morphology and sensorial perception

**DOI:** 10.1038/srep28316

**Published:** 2016-06-20

**Authors:** Jakub Prokop, Martina Pecharová, Dong Ren

**Affiliations:** 1 Charles University in Prague, Faculty of Science, Department of Zoology, Viničná 7, CZ-128 44, Praha 2, Czech Republic; 2 Key Lab of Insect Evolution & Environmental Changes, College of Life Science, Capital Normal University, Beijing 100048, PR China

## Abstract

Megasecoptera are insects with haustellate mouthparts and petiolate wings closely related to Palaeodictyoptera and one of the few insect groups that didn’t survive the Permian-Triassic mass extinction. Recent discovery of *Brodioptera sinensis* in early Pennsylvanian deposits at Xiaheyan in northern China has increased our knowledge of its external morphology using conventional optical stereomicroscopy. Environmental scanning electron microscopy (ESEM) of structures, such as antennae, mouthparts, wing surfaces, external copulatory organs and cerci have shed light on their micromorphology and supposed function. A comparative study has shown an unexpected dense pattern of setae on the wing membrane of *B. sinensis*. In addition, unlike the results obtained by stereomicroscopy it revealed that the male and female external genitalia clearly differ in their fine structure and setation. Therefore, the present study resulted in a closer examination of the microstructure and function of previously poorly studied parts of the body of Paleozoic insects and a comparison with homologous structures occurring in other Palaeodictyopteroida, Odonatoptera and Ephemerida. This indicates, that the role and presumptive function of these integumental protuberances is likely to have been a sensory one in the coordination of mouthparts and manipulation of stylets, escape from predators, enhancement of aerodynamic properties and copulatory behaviour.

Megasecoptera is a small group of Late Paleozoic phytophagous insects having haustellate type mouthparts in the form of a rostrum with elongated stylets and permanently outstretched basally narrow wings with corrugated longitudinal veins and generally few cross-veins. This group belongs to the extinct Palaeodictyopteroida, are uncertain systematic position and either an ingroup of Palaeoptera or sister-group of Neoptera^1^,^2^,^3^. The family Brodiopteridae are restricted to the Namurian stage with one of its members, *Brodioptera stricklani*, the oldest Megasecoptera and at the same time one of the earliest winged insects (Pterygota) recorded close to the boundary between Mississippian and Pennsylvanian^4^. Recent discovery of the genus *Brodioptera* in Early Pennsylvanian deposits in China provides evidence for faunal exchange between Euroamerica and Northern China during the Bashkirian^5^. *Brodioptera sinensis* was described on the basis of a well preserved series of specimens in various aspects of preservation, which revealed intra-specific variability in wing venation^6^. Moreover, the reconstructed species shows details of its morphology, like haustellate mouthparts with conspicuous elongated stylets, wings with a well preserved pattern of venation and male and female external genitalia that were previously poorly documented or unknown in these insects (see Fig. 1). Therefore, this material offers an excellent opportunity for a detailed study of the micromorphology of certain structures using ESEM and to use this information to determine the likely function of similar structures in related taxa.

Scanning electron microscopy (SEM) has been used to study surface microstructures of arthropods for almost half a century. However, it is rather rarely used for studying Paleozoic insect fossils, with a few exceptions, such as searching for pollen grains or spores in their gut contents, studying the micromorphology of delicate structures, chaeototaxy and phoretic organisms^7^,^8^,^9^,^10^. The poor state of the majority of imprint fossils damaged by postmortal deformations makes their study particularly difficulty and often end in failure. These techniques have been more widely used in studies of fossils from younger strata, such as Mesozoic and Cenozoic amber inclusions and exceptionally preserved compressed fossils^11^,^12^,^13^,^14^. The broader application to the study of scarce insect fossils was accelerated recently with use of ESEM, which makes it possible to study uncoated specimens using this non-invasive technique^15^.

Thus, this study presents a more detailed and clearer view of surface microstructure of certain parts of the body of a megasecopteran, *B. sinensis*, using the extensively and well documented material from the Late Carboniferous in northern China^6^.

## Results and Discussion

### Head structures, in particular antennae and mouthparts

The head is hypognathous, relatively small and triangular in shape with elliptical bulging eyes (ce) (Fig. 2a). Antennae filiform, generally incompletely preserved and nearly reaching the tip of the rostrum (CNU–NX1–602a), with enlarged scape (sc) and short pedicel (pe), both poorly delimited and a long multi-segmented flagellum consisting of short elliptical flagellomeres (fl) (Fig. 2b). These long antennae were either held wide spread in flight, which would greatly reduce their air speed, or positioned closely along the sides of the rostrum as in several specimens in which these structures are fossilized. Mouthparts of haustellate type with a rostrum; the beak, consist of a pair of slender long markedly sclerotized mandibular stylets (md) (Fig. 2b,c) and paired less sclerotized stout multi-segmented maxillary palps (mp) basally connected to the maxillae underneath the md stylets, the microstructure on consists of scattered sensory setae on up to 180 μm of their length (Fig. 2c,d). This insect probably used these setae as mechanoreceptors for coordinating its mouthparts and manipulation of stylets while feeding. The two pairs of aforementioned stylets dorsally cover the labium (lb) that consists of a pair of lobes (Fig. 2c). The head capsule is usually distorted due to taphonomy, but a triangular labrum (lm) and poorly preserved domed trapezoidal clypeus (cl) can be distinguished (Fig. 2a,b).

This type of unique mouthparts with a prominent rostrum is considered as a synapomorphy for members of the Palaeodictyopteroida, which includes the orders Palaeodictyoptera, Megasecoptera, Permothemistida and Diaphanopterodea^1^,^2^,^4^,^16^. However, the mouthparts of these fossils are poorly preserved and hence only a few taxa have been studied in detail, for instance the diaphanopterodean *Permuralia maculata* known from the Early Permian in Russia^16^,^17^,^18^,^19^, and palaeodictyopteran *Eugereon boeckingi* (Eugereonidae) known from the Late Carboniferous in Germany and *Monsteropterum moravicum* (Homoiopteridae) from the Early Permian in the Czech Republic^20^,^21^ among others. The mouthparts of *B. sinensis* seem to correspond particularly well with those of *P. maculata* in having a distinctly shorter triangular labrum, a pair of closely aligned strongly sclerotized mandibulary stylets and broad and weakly sclerotized maxillary stylets. The rostrum of *B. sinensis*, however, is markedly longer than that of *P. maculata*, which may indicate it fed on different type of plant. Interestingly, another megasecopteran, with a relatively short and stout beak, *Sinopalaeopteryx olivieri* (Aykhalidae) is also known from Xiaheyan, which is evidence of the evolution of different types of herbivory in this group of insects during the Bashkirian^22^. In this context, it is noteworthy that specific piercing and sucking damage caused by the stylets of Palaeodictyopteroida to marattialean tree ferns and to Cordaite seeds are recorded^16^,^23^,^24^,^25^. Nevertheless, such record from Xiaheyan locality has not been reported so far.

Material examined: Nos. CNU–NX1–600a, b (holotype); CNU–NX1–617 (paratype); CNU–NX1–602a,b; CNU–NX1–609a,b; CNU–NX1–615a,b; CNU–NX1–621a,b; CNU–NX1–632; CNU–NX1–651a,b.

### Wing surface structures

The shape and poorly developed wing musculature of the wings of Megasecoptera indicate that they were adapted for slow flight or even hovering^26^. The wing membrane of *B. sinica* is covered by irregularly scattered setae with a few also on the veins. There is a marked decrease in the density of setae from the base to the apex of a wing. All these setae, are on basal circular sockets (Fig. 3c,d), nearly straight and structurally similar, with a maximum size of about 100 μm and, therefore, considered to be macrotrichia (Fig. 3a–d). They probably functioned as mechanoreceptors. The surface structures on insect wings, such as small bristles, scales and setae are widely studied in recent insects, especially in terms of their association with flight ability. These structures are active during flight in members of the Odonata, Diptera, Hymenoptera and Lepidoptera^27^,^28^,^29^. The setae on the wing membrane cause microturbulences and possibly decrease friction during flight by creating a boundary layer between the air stream and wing surface^30^. That is, these setae could provide a flying insect with enhanced lift and reduced drag, or alternatively the setae are hydrophobic as they are much longer and more clustered in teneral adults or potentially subimago than in the adults. Similar structural differences in microstructure of wings of adult and subimago modern mayflies are reported by several authors^31^. However, these setae on the wing membrane of modern Ephemeroptera are microtrichia and distinctly smaller in size. Thus, the suggestion that the function of these structures is hydrophobic (microtrichia) in megasecopterans is not well supported. Particularly, because it implies an aquatic lifestyle of the nymphs of these insects, which is currently not widely accepted, and supports the original idea^32^,^33^,^34^. The record of their immature stages is rather poor and available specimens entirely known from the Carboniferous ironstone nodules as members of Mischopteridae do not show the mentioned micro structural details which could enlighten their lifestyle unambiguously^35^. The specialized type of mouthparts of nymphs and their articulated wing pads in slightly expanded position from the thorax support hypothesis that they had a similar diet and habitat preference as adults. Nevertheless, it is not possible to exclude the possibility that *B. sinica* had two types of setae on its wings, as is the case in modern lacewings, like *Micromus tasmaniae* (Hemerobiidae), and the tiny microtrichia cannot be observed due to taphonomy^36^ (Figs 3 and 4).

Our observations on wing surface microstructure of various specimens of *B. sinica* indicates two different stages: a teneral adult or potentially subimago (CNU–NX1–605, CNU–NX1–609, CNU–NX1–632) with wings without darkly coloured apices, which are often creased with distinctly longer and clearly more densely clustered macrotrichia, possibly having a hydrophobic function, and an imago with darkly coloured wing apices, setae on wing membrane distinctly shorter and sparsely clustered. Moreover, this is supported by females with the ovipositor in the teneral adult or subimago stage slender and always hidden in the third pair of valvulae, while in the imago the ovipositor is broad and the first and second pair of valvulae preserved in an exposed position and separate from the third pair of valvulae.

The wing membrane of Megasecoptera is generally hyaline with rarely any well-developed macrotrichia as in members of the family Bardohymenidae^37^. The venation of *Actinohymen russelli* (Bardohymenidae) bears setal sockets in rows on the main longitudinal veins CA+CP, ScP and R^38^. Prominent serrations or knob–like elliptical protuberances (tubercles) are also documented on the veins on the anterior wing margin of Brodiidae, as in *Brodia priscotincta* and *Eubrodia dabasinskasi*^39^. Bolton considers these spinules to be modifications of long hairs^40^, “macrotrichia”, which supports the view of Tillyard^41^. Corresponding structures are known in other members of Palaeodictyopteroida (e.g., Anchineuridae, Namuroningxiidae (see Fig. 4a), Protohymenidae) and also in some members of recent insects, like Odonata, etc^42^,^43^,^44^. Moreover, there are prominent spines projecting apically on the basal part of the margin of the hind wing in Brodiidae^39^. Kukalová-Peck records the common presence of macrotrichia on wings of members of Palaeodictyoptera^45^, nevertheless, the evidence for this is weak. Macrotrichia on the membrane are not widespread among megasecopterans, as demonstrated for *Namuroptera minuta* (Aykhalidae, see Fig. 4b), which is known from the same locality as *B. sinensis* and lacks such setae on its wing membrane^22^. Hence, this disparity in wing surface microstructures in Megasecoptera was present since at least the Late Carboniferous and their function is probably related to their flight ability rather than hydrophobic.

Material examined: CNU–NX1–600a,b (holotype); CNU–NX1–601a,b; CNU–NX1–605; CNU–NX1–609a,b; CNU–NX1–621a,b; CNU–NX1–632.

### Abdomen, in particular external genitalia, cerci and their microstructures

External copulatory organs of Carboniferous insects are rarely recorded especially for both sexes in several specimens. Therefore, their fine structure revealed by ESEM is described in this section. The abdomen of *B. sinica* is 10-segmented with apex bearing a pair of long multi-segmented and rather stout cerci. The cerci are covered with prominent protruding setae up to 500 μm long arranged in rings along the posterior edge of each segment. We think that these setae are trichoid sensillae, which are common in modern insects and most probably function as tactile sensory setae or air movement receptors for control of yaw stability in flight (see Fig. 5). It is likely that these setae enable them to detect the lunging movement of a predator and immediately escape by running or flying away. Novokshonov and Willmann describe segmented basally stout cerci bearing long setae arranged along the posterior margin in the diaphanopterodean *Asthenohymen uralicum* (Asthenohymeniodae) from the Early Permian in Chekarda in the Central Urals^46^.

Male external genitalia consist of enlarged forceps base (styliger, st) with the slender posteriorly situated forceps (fc) curving distally (Figs 6 and 7b). Forceps are at least two segmented and reach the tip of the abdomen as was described in our previous study, but re-examination of specimen CNU–NX1–601b using ESEM revealed the possible presence of a short additional terminal segment (Fig. 6b). Penis clearly consists of two penial lobes (pl) slightly enlarged basally and straight for sperm transfer (Fig. 6a,c). The ESEM study also indicates the apices of penial lobes are more slender (possibly titillator processes) than reported in our previous study using light stereomicroscopy^6^. It is likely that there are setae on the surface of the penial lobes and a cluster of terminal setae at their apices (Fig. 6c) but we are unable to confirm this due to processes that occurred during taphonomy. Homologous structures are recorded in *Permohymen schucherti* (Protohymenidae) from the Lower Permian in Kansas^47^, which bear a pair of two segmented claspers (forceps) and two penial lobes that are strikingly similar to those of *B. sinensis*. However, on the basis of our re-examination of specimen MCZ 3819b these penial lobes are distinctly shorter and have stouter terminal appendages than *B. sinensis*. The forceps (gonostyli) are covered with tactile setae that are probably mechanoreceptors, however their distal segments bear densely clustered setae. Another brodiopterid, *B. stricklani*^48^, known from the Bashkirian in North America has male genitalia that are very similar with the lateral elongated claspers acting as forceps, but a closer comparison of their segmentation is not possible because of their poor state of preservation. Furthermore, our re-examination of Permian *Protereisma permianum* (Permoplectoptera: Protereismatidae) and *Misthodotes obtusus* (Permoplectoptera: Misthodotidae) revealed that the structures of male external genitalia bearing the enlarged forceps basis with a pair of five segmented forceps pointed apically and elongated slender apices of the penial lobes are homologous^49^. Thus, this study of morphology, including surface microstructures, confirms that the external male copulatory organs of Megasecoptera and Ephemerida are very similar as previously suggested^48^,^50^ and others. Furthermore, our comparison was of several well documented members of the order Diaphanopterodea in which the structures of the external male genitalia and other body characters are homologous, but at rest the wings of which are positioned along the abdomen. The best studied species is *P. maculata*, for which the male external copulatory organs are reconstructed and interpreted differently^17^,^19^. Our comparison is supported by the re-examination of series of specimens adopting the more conservative view^19^. In the poorly preserved *Permuralia* the corresponding structures are two segmented forceps (gonocoxae and gonostyli) and well separated and straight penial lobes. Similarly, male external copulatory structures bearing long two segmented forceps with tubercles on inner part of the gonostyli are also described in *Asthenohymen uralicum*. Finally, the comparison of *B. sinensis* to the corresponding primary male copulatory structures known in *Namurotypus sippeli* (Meganisoptera: Namurotypidae) as one of early diverging group of Odonatoptera revealed the presence of a pair leaf-like segmented gonopods and paired penial lobes which are regarded as synapomorphy of Palaeodictyopteroida, Ephemeropterida and Odonatoptera^51^. Considering the length of distal abdominal segments and position of forceps in *B. sinensis* in contrast to extant mayflies we assumed that high flexibility of these segments allowing copulatory position. Interestingly, the hypothesis of indirect copulation behavior and deposition of spermatophores as proposed for Namurotypidae by Bechly^51^ seems to be unlikely for *Brodiptera* due to striking resemblance of distally pointed forceps with modern mayflies and also the presence of clustered sensory structures.

Material examined: CNU–NX1–617 (paratype); CNU–NX1–601a,b; CNU–NX1–602a,b; CNU–NX1–610a,b.

Female external genitalia consist of a prominent ovipositor with two pairs of cutting valvulae (V1, V2), which extend backwards to the posterior edge of the 10th abdominal tergite, and markedly larger sheathing valvulae (V3) (Figs 7a and 8). Cutting valvulae (V1) are nearly straight with swollen bases that are connected to the basal plate of the ovipositor (bp) and the sternite of the 8^th^ segment. Anterior part of bp is markedly concave between apophyses on the medial and lateral apodemes of the ovipositor basal plate (b,a) and its posterolateral part most probably represented by the anterior parts of the gonangulum (gon). Dorsal edge of V1 forming a longitudinal groove (aulax) and forms a sliding joint (olistheter), which enables the first and second pair of the valvulae to be moved in opposite directions, like the saw in the endophytic ovipositor of zygopteran Odonata^52^. The longitudinal ridge like keel (rhachis) on the ventral edge of V2 fit into the aulax (Fig. 8a,e). The surface of the distal part of V1 with 9–10 (11?) oblique prominent hook like ridges and approximately seven ridges on V2, probably function as a saw (Fig. 8f). The third pair of sheathing valvulae (V3) enclose the cutting valvulae when in a resting position. Sheathing valvulae are broader than the cutting valves and their surface bear scattered long setae (Fig. 8a,c). Surprisingly, the preservation is so good that it is possible to see in part the endoskeleton of V3, with two apophyses, aAp and pAp (see Fig. 7a). On the other hand, the detailed examination of the apical part of valvulae V3 of several specimens did not confirm the presence of a stylus as in *Permuralia* (Diaphanopterodea), *Monstropterum moravicum* (Paleodictyoptera) or modern Odonata^17^,^19^,^21^. The morphology of the ovipositor is unambiguously of endophytic type and in many aspects is comparable to that in modern damselflies (Odonata: Zygoptera), but sheathing valvulae (V3) clearly lack apical denticles in the form of a carina and an apical stylus^52^. Nevertheless, the experimental studies with extant endophytic Odonata shown that removal of styli has influenced position of eggs in egg sets which has to be considered as complex oviposition behavior driven by sensory organs on styli^53^. We assumed that such functionally sophisticated system of regular egg patterning in clutch evolved in some groups from less efficiently arranged oviposition. Surprisingly, the fossil record of endophytic oviposition can be traced back to the Pennsylvanian with the earliest evidence of scars as endophytic oviposition cavities on stem of *Calamites cistii* (Sphenophyta) known from Graissessac Basin in France reflecting rather irregular pattern of eggs in clutch^54^. While the fossil record of oviposition scars documented from younger strata (Permian) show generally more regular patterning of oviposition^55^. The recent discovery of endophytic oviposition in form of egg cavities arranged in longitudinal rows or zigzag configuration on leaf of *Cordaites* from the Pennsylvanian of Wettin member in Saale Basin strongly resambles the arrangement of eggs known in damselflies of Coenagrionidae (Zygoptera) and therefore the oviposition probably was caused by a member of the extinct odonatopteran suborder Archizygoptera^56^. So far the rich plant fossils from Xiaheyan locality given any evidence of oviposition scars yet and thus we cannot be sure to which plant *Brodioptera* layed its eggs.

Interestingly, the reduction of stylus on sheathing valvulae is also considered in case of stem odonatopteran *Erasipteroides valentini* (Erasipteridae) from Namurian of Hagen-Vorhalle in Germany, but its extreme length support rather endosubstratic oviposition^51^. On the other hand, the most primitive known fossil dragonflies Eugeropteridae had a short ovipositor.

Material examined: CNU–NX1–600a, b (holotype); CNU–NX1–613a; CNU–NX1–624a,b; CNU–NX1–651a,b.

## Conclusions

For the first time, microstructures on the integumental surface were studied comprehensively on a large number of fossil specimens of a Megasecopteran species. In spite of the limitations imposed by the poor state preservation due the processes occurring during taphonomy this study revealed details of their microstructure and how selected body structures functioned in a Late Carboniferous insect, which lived approximately 317 Mya.

Our reconstruction of *B. sinensis* revealed it had a hypognathous head with prominent haustellate mouthparts in the form of a rostrum consisting of a basally short triangular labrum, two pairs of mandibulary and maxillary stylets, stout multi-segmented maxillary palps extending beyond the tip of stylets and covered with large sensory setae, and a labium with a pair of lobes underneath the stylets. We assume that these specialized piercing and sucking mouthparts were adapted for feeding on the spores of an unknown plant, like tree ferns or Cordaites. Antennae were filiform, reaching the tip of the stylets. Thorax with walking legs, narrow prothorax, meso- and metathorax approximately equal in size bearing two pairs of homonomous outstretched wings. The surface microstructure on the wings consists of irregularly scattered macrotrichia on the membrane and veins, which markedly decrease in density from the base to the apex of the wing, which possibly functioned as mechanoreceptors. Furthermore, it is likely that the fossils are of two different stages: teneral imago or subimago with hyaline wings with setae more clustered and a slender ovipositor always hidden in a third pair of valvulae and adults with dark coloured wing apices, less clustered setae on wings and ovipositor with the first two pairs of valvulae always more exposed than the third pair. Abdomen is 10-segmented and bears a pair of long multi-segmented cerci covered with protruding tactile sensory setae as in other members of the Palaeodictyopteroida. The most interesting feature are external copulatory organs that are rarely recorded in Carboniferous insects, which are present both sexes. Male genitalia consist of enlarged basal forceps (styliger), posteriorly slender two-segmented forceps curved distally and two basally enlarged penial lobes with slender apices for sperm transfer. We confirmed the presence of scattered long setae on the forceps and of a cluster of setae on the slender apices of the penial lobes. Female genitalia consist of an endophytic ovipositor with two pairs of cutting valvulae (V1 and V2) and a pair of enlarged sheathing valvulae (V3) covered with a scattering of long setae. Surfaces of the distal parts of V1 and V2 with prominent hook like ridges are used for cutting plant tissue. Dorsal edge of V1 forming a longitudinal groove (aulax), which forms a sliding joint (olistheter) that enables the first and second pair of valvulae to move in opposite directions. The longitudinal ridge like keel (rhachis) on the ventral edge of V2 fits into the aulax as in modern endophytic zygopteran dragonflies^52^. Surprisingly, the third pair of valvulae lacks a stylus, which is well developed in few members of Diaphanopterodea and Palaeodictyoptera. Thus, in the Late Carboniferous this difference in the morphology of the ovipositor and associated behaviour was already established in the Palaeodictyopteroida. Nevertheless, a comparison of this trait in members of the Palaeodictyopteroida is not possible because there are only a few taxa with a third pair of valvulae.

**Figure 1:**
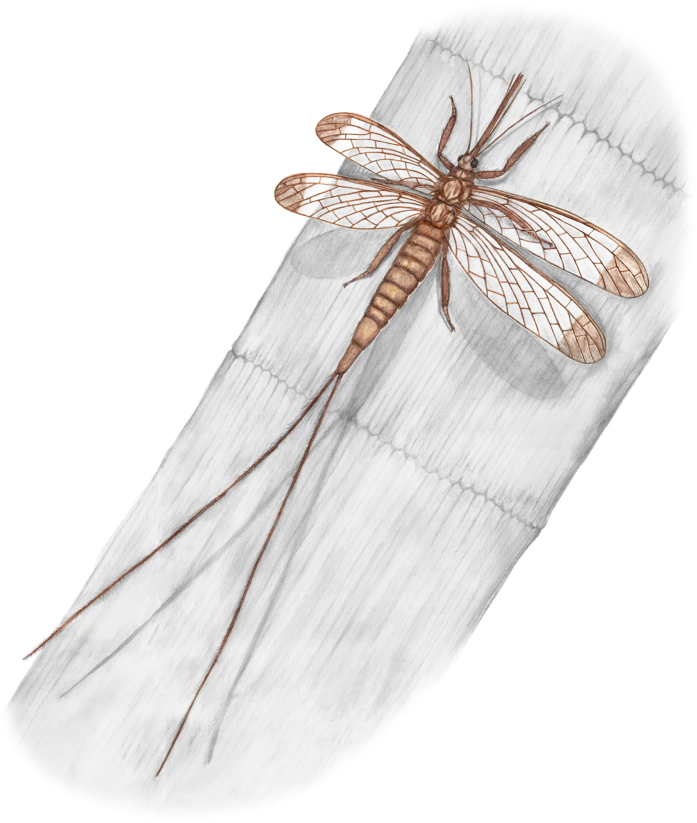
*Brodioptera sinensis*, adult female, reconstruction of habitus based on series of the specimens as resting on sphenophyte stem *Calamites* sp. (Calamitaceae), wing span cca 46 mm, Late Carboniferous, China (drawn by MP).

**Figure 2:**
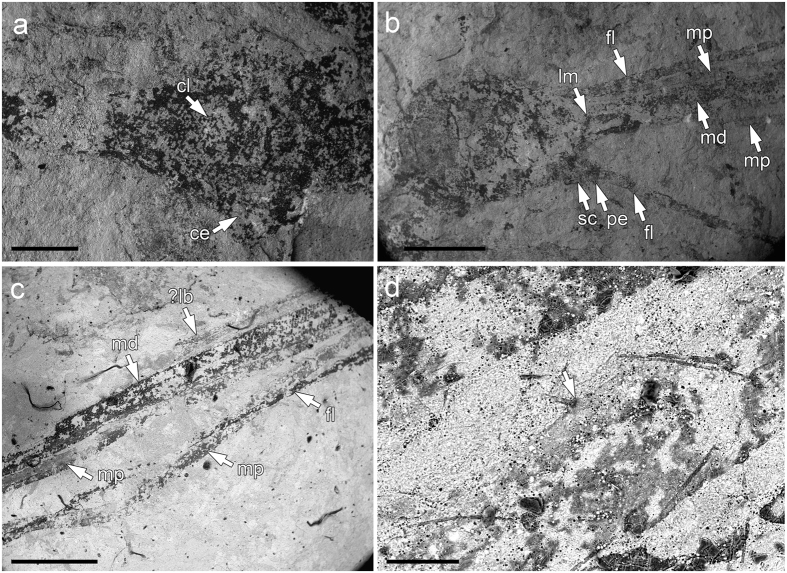
*Brodioptera sinensis*, scanning electron micrographs of head structures, Capital Normal University, Beijing, China. (**a**) Surface of CNU–NX1–609 (**b**) CNU–NX1–602 (**c**,**d**) stylets with close ups surface of setae CNU–NX1–651. Arrows indicate ce - compond eye, fl - flagelum, lb - labrum. Scale bars 500 μm (**a**), 1000 μm (**b,c**), 100 μm (**d**).

**Figure 3:**
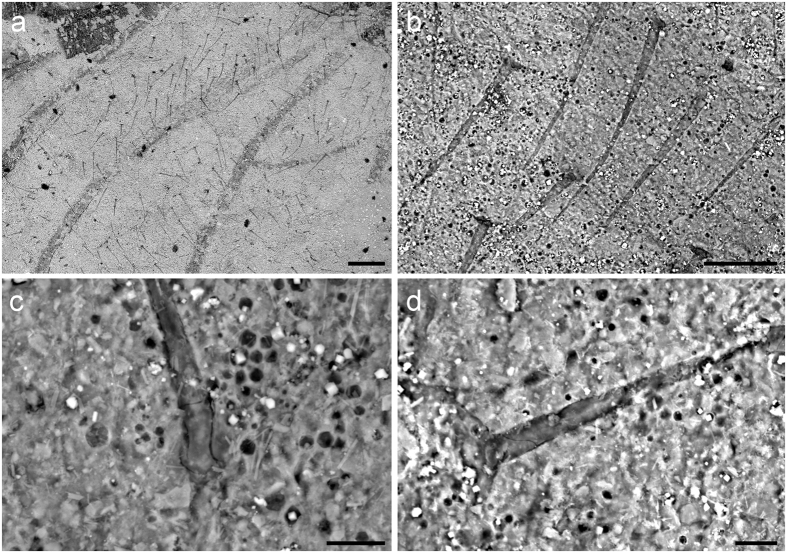
*Brodioptera sinensis*, scanning electron micrographs of wing structures, Capital Normal University, Beijing, China. (**a**) Surface of hindwing anal area with scattered setae CNU–NX1–632. (**b–d**) Detail of setae CNU–NX1–632. Scale bars 200 μm (**a**), 50 μm (**b**), 10 μm (**c,d**).

**Figure 4:**
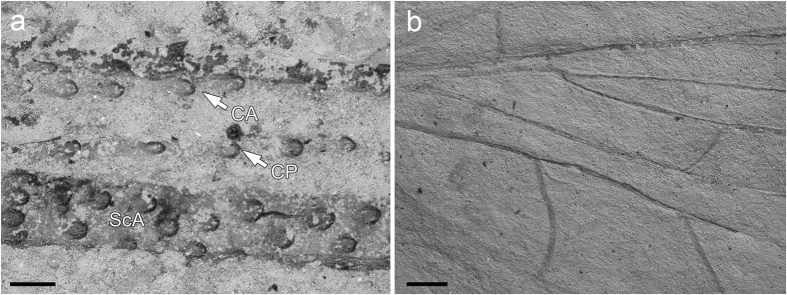
*S*canning electron micrographs of wing structures, Capital Normal University, Beijing, China. (**a**) *Namuroningxia elegans* (Namuroningxiidae) with knob like tubercles located on the veins CA, CP and ScA, CNU–P–NX2006001. (**b**) *Namuroptera minuta* (Aykhalidae), surface of wing basal part CNU–NX1–646. Scale bars 200 μm (**a,b**).

**Figure 5:**
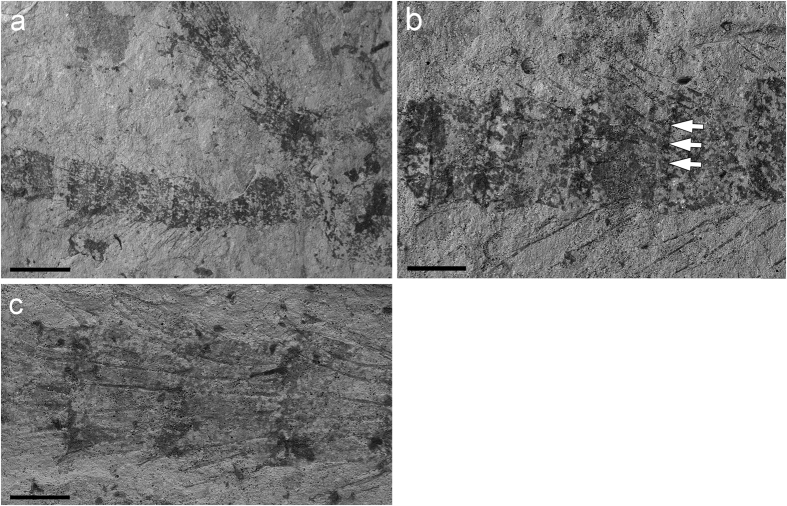
*Brodioptera sinensis*, scanning electron micrographs of cerci, Capital Normal University, Beijing, China. (**a**) Apex of abdomen with basal part of cerci CNU–NX1–602a. (**b**) Detail of cerci with protruding setae CNU–NX1–602a. (**c**) Detail of cerci with protruding setae CNU–NX1–600b. Arrows indicate bases of setae surround the posterior edge of segments. Scale bars 500 μm (**a**), 200 μm (**b,c**).

**Figure 6:**
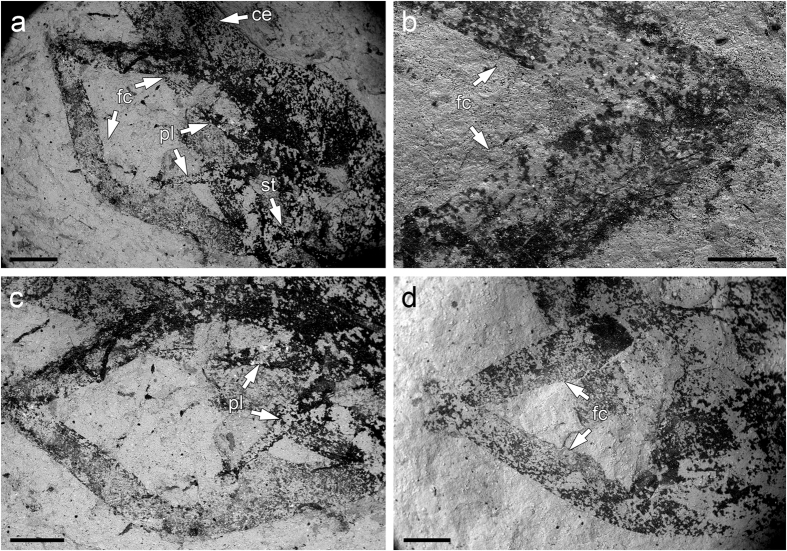
*Brodioptera sinensis*, scanning electron micrographs of male external genitalia, Capital Normal University, Beijing, China. (**a**) Forceps, penial lobes and styliger CNU–NX1–601a. (**b**) Detail of forceps apices with protruding setae CNU–NX1–601a. (**c**) Forceps and penial lobes CNU–NX1–601a. (**d**) Forceps CNU–NX1–610b. Arrows indicate cerci (ce), forceps (styli) (fc), penial lobes (pl) and styliger (st). Scale bars 600 μm (**a**), 200 μm (**b**), 500 μm (**c,d**).

**Figure 7:**
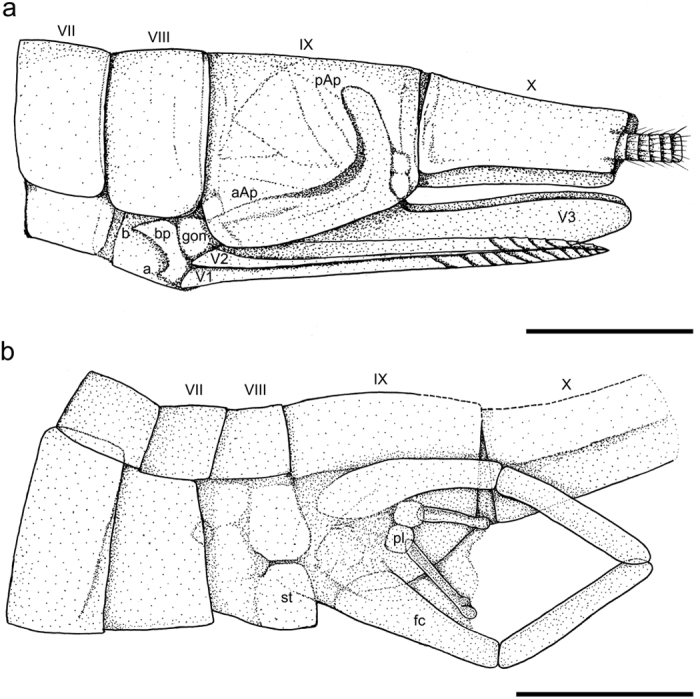
*Brodioptera sinensis*, line drawings with partly reconstructed endoskeleton (drawn by MP). (**a**) Distal part of abdomen bearing female external genitalia based on specimen CNU–NX1–651b. (**b**) Distal part of abdomen bearing male external genitalia based on paratype CNU–NX1–601a. Abbreviations: a/b – medial/lateral apodeme of basal plate of ovipositor, aAp/pAp – anterior/posterior apophysis of V3; au – aulax, bp – basal plate of ovipositor (lamina valvarum), ce – cerci, fc – forceps, gon – gonangulum, pl – penial lobes (penes), V1/V2/V3 – first/second/third valvulae of ovipositor, st – styliger (forceps base). Scale bars 3 mm (**a**), 2 mm (**b**).

**Figure 8:**
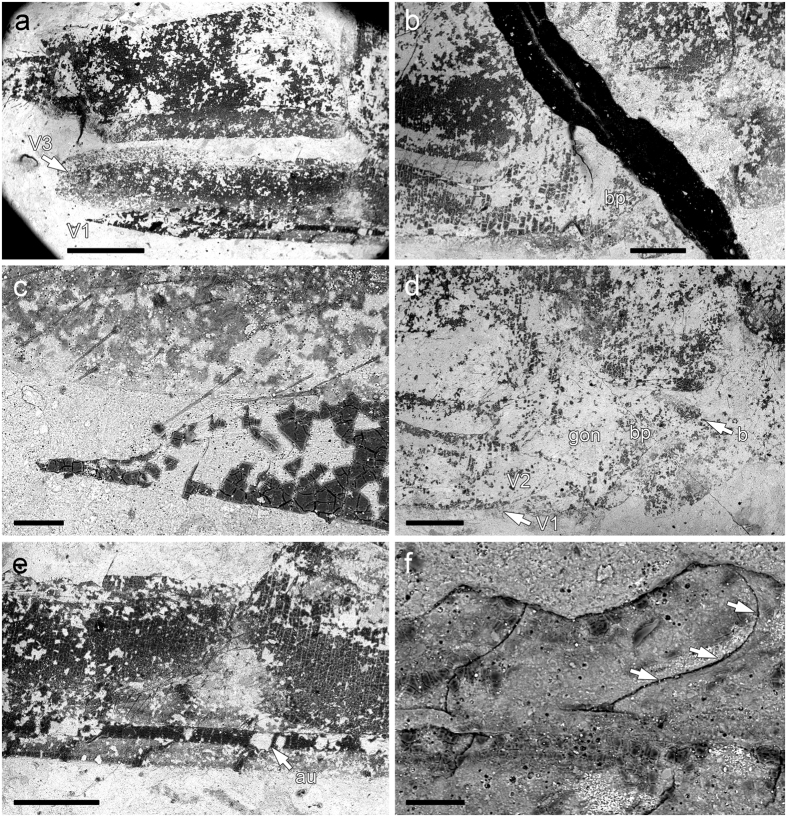
*Brodioptera sinensis*, scanning electron micrographs of female external genitalia, Capital Normal University, Beijing, China. (**a–e**) Distal part of abdomen bearing female external genitalia CNU–NX1–651b. (**c**) Detail of ensheathing valvulae V3 bearing scathered setae. (**d**) Detail of ovipositor base with basal plate of ovipositor and gonangulum. (**e**) Detail of medial part of ovipositor with longitudinal groove (aulax) allowing a sliding joint (olistheter). (**f**) Detail of ovipositor valvulae V2 with prominent hook like ridges, CNU–NX1–624a. Scale bars 1000 μm (**a,b**), 100 μm (**c**), 500 μm (**d,e**), 50 μm (**f**).

Finally, it is likely that *B. sinensis* was a slow flying insect with a head bearing a long beak held in an hypognathous position, filiform antennae reaching the tip of the mouthparts and widely spread cerci as in recent mayflies (see Fig. 1). Nevertheless, flight in the dense hyperoxic Carboniferous atmosphere was easier in terms of the energy required^57^.

## Methods

### Material and analysis

The material was a complete series attributed to *B. sinensis* consisting of 54 compressed fossils, including the holotype and paratype, ranging from fragmentary isolated wings to nearly complete specimens. Material was initially sorted mainly based on the presence of fine structures and quality of preservation. Twelve specimens were selected for environmental scanning electron microscopy and others were also examined for specific structures. All specimens examined in this study are housed in the Key Laboratory of Insect Evolution and Environmental Changes at Capital Normal University (prefix CNU-) in Beijing (China). Conventional study of the external morphology of all the available specimens using optical stereomicroscopy including the taxonomy was published^6^. Scanning electron micrographs of uncoated specimens were obtained using an environmental electron microscope Hitachi S-3700N (Hitachi Ltd, Chiyoda, Tokyo, Japan) at an accelerating voltage of 15 kV with a turntable sample holder at the Department of Paleontology, National Museum in Prague.

Terminology used for general insect morphology^58^ and for external genitalia^59^,^60^. The terminology used for the description of the ultrastructure of setae^30^,^58^. The terms of macro- and microtrichia are classified based on their length (under and above 5 μm), based on presence vs. absence of setal socket and they are collectively referred as hairs. Large processes on wing are called bristles and tactile setae are called trichoid sensilla^30^. Naturally, without application of transmission electron microscopy for ultrastructure there is still some uncertainty to discern between these kinds of cuticular structures.

Abbreviations used for morphological structures are: ce – compound eyes, cl – clypeus, fl – flagelum, md – mandibulary stylets, mp – maxilary palps, mx – maxilary stylets, lb – labium, lm – labrum; abdomen: a/b– medial/lateral apodeme of basal plate of ovipositor, aAp/pAp – anterior/posterior apophysis of V3; au – aulax, bp – basal plate of ovipositor (lamina valvarum), ce – cerci, fc – forceps, gon – gonangulum, pl – penial lobes (penes), V1/V2/V3 – first/second/third valves of ovipositor, st – styliger (forceps base). The wing venation in general follows^61^ and the nomenclature is adopted. Wing venation abbreviations: A1/A2 – first/second anal vein, CA/ CP – costa anterior/posterior, CuA/CuP – cubitus anterior/posterior, MA/MP – media anterior/posterior, RA/RP – radius anterior/posterior, ScP – subcosta posterior.

### Outcrop location and age

All the insect specimens examined in this study came from Xiaheyan in Zhongwei County in the Ningxia Autonomous Region of northwestern China. Insects are preserved as compressed fossils in terrestrial facies of the Tupo Formation dated to Namurian B/C (early Bashkirian), which are biostratigraphically correlated with deposits in Europe, North America and Russia^62^,^63^. The paleoenvironment in which these sediments were deposited is interpreted as marine-lagoonal with tidal flats and marshlands^64^.

## Additional Information

**How to cite this article**: Prokop, J. *et al*. Hidden surface microstructures on Carboniferous insect *Brodioptera sinensis* (Megasecoptera) enlighten functional morphology and sensorial perception. *Sci. Rep.* **6**, 28316; doi: 10.1038/srep28316 (2016).
